# Preventing opioid use among justice-involved youth as they transition to adulthood: leveraging safe adults (LeSA)

**DOI:** 10.1186/s12889-021-12127-3

**Published:** 2021-11-20

**Authors:** Danica Kalling Knight, Yang Yang, Elizabeth D. Joseph, Elaine Tinius, Shatoya Young, Lillyan T. Shelley, David R. Cross, Kevin Knight

**Affiliations:** Texas Christian University, 2901 University Drive, TCU Box 298921, Fort Worth, TX 76129 Texas USA

**Keywords:** Trust-based relational intervention, Prevention intervention for opioid use, Hybrid type I design, Trauma-informed care, Justice-involved youth

## Abstract

**Background:**

Juvenile justice (JJ) youth are at high risk of opioid and other substance use (SU), dysfunctional family/social relationships, and complex trauma. The purpose of the Leveraging Safe Adults (LeSA) Project is to examine the effectiveness of Trust-Based Relational Intervention® (TBRI®; leveraging family systems by providing emotional and instrumental guidance, support, and role modeling) in preventing opioid and other SU among youth after release from secure residential facilities.

**Methods:**

An effectiveness-implementation Hybrid Type 1 design is used to test the effectiveness of TBRI for preventing non-medical use of opioids among JJ-youth (delayed-start at the site level; a randomized controlled trial at participant level) and to gain insight into facility-level barriers to TBRI implementation as part of JJ re-entry protocols. Recruitment includes two samples (effectiveness: 360 youth/caregiver dyads; implementation: 203 JJ staff) from nine sites in two states over 3 years. Participant eligibility includes 15 to 18-year-olds disposed to community supervision and receiving care in a secure JJ facility, without active suicide risk, and with one caregiver willing to participate. Effectiveness data come from (1) youth and caregiver self-report on background, SU, psychosocial functioning, and youth-caregiver relationships (Months 0, 3, 6, 12, and 18), youth monthly post-release check-ins, and caregiver report on youth psychological/behavioral symptoms, and (2) JJ facility records (e.g., recidivism, treatment utilization). Fidelity assessment includes post-session checklists and measures of TBRI strategy use. Collected four times over four years, implementation data include (1) JJ staff self-report on facility and staff characteristics, use of trauma-informed care and TBRI strategies, and (2) focus groups (line staff, leadership separately) on use of trauma-informed strategies, uptake of new interventions, and penetration, sustainment, and expansion of TBRI practices.

**Discussion:**

The LeSA study is testing TBRI as a means to empower caregivers to help prevent opioid use and other SU among JJ-youth. TBRI’s multiple components offer an opportunity for caregivers to supplement and extend gains during residential care. If effective and implemented successfully, the LeSA protocol will help expand the application of TBRI with a wider audience and provide guidance for implementing multi-component interventions in complex systems spanning multiple contexts.

**Trial registration:**

ClinicalTrials.govNCT04678960; registered November 11, 2020; https://clinicaltrials.gov/ct2/show/NCT04678960.

## Background

Across the US, an estimated 10.8 million people misuse prescription opioid analgesics (including prescription opioids and illicit opioids, such as heroin and illicitly made fentanyl and related analogs) [1]. Sixty percent of overdoses are attributed to opioids [2, 3], which are now the 5th leading cause of accidental death [4]. Experimentation and regular use of opioids escalate in the late teens and early 20s [5–8]. Nationally, 16.6 million youth aged 14–17 are involved in the US juvenile justice (JJ) system [9], making it a critically important intervention opportunity [10]. Substance use (SU) among JJ youth is associated with deviant behavior and recidivism [11, 12], physical and mental health (MH) problems [13–16], and places youth at higher risk for non-medical use of opioids [17].

SU risk is heightened among youth who are transitioning back into their communities after a period of residential detainment or treatment and especially among those who have a history of adverse childhood experiences (ACEs) leading up to JJ involvement [18]. Living arrangements can be tentative, family support for rehabilitation unstable, the means to pursue vocational and/or educational goals may be sparse, and families may have limited access to (or knowledge of) available supports. Many youth return to behaviors, thinking patterns, and social groups that are familiar and negative [19]. It is therefore imperative that opioid prevention interventions assist youth (particularly those with childhood trauma) in developing relationships with adults who serve as positive guides and mentors through their transition into young adulthood.

Trauma is particularly problematic among JJ youth [20]. Adverse childhood experiences underlying trauma are risk factors to MH disorders [21, 22]; earlier onset of opioid and other SU, drug overdose [23]; and use in young adulthood [24, 25]. Furthermore, complex trauma (i.e., chronic and repeated exposure) is associated with offending and recidivism [26] [27]. Because protective relationships with adults can moderate the relationship between ACEs and SU [20] [28], addressing trauma as part of interventions to prevent opioid initiation is critically important [21].

Strong family relationships [29], healthy attachment bonds [30], and support systems that foster positive coping and emotional autonomy [31] are robust protective factors against SU. However, many families are ill-equipped to provide effective guidance and support, lacking knowledge and experience in how to interact in supportive and nurturing ways, and effectively direct youth toward healthy independence and autonomy [32]. Interventions are needed that systemically address risk factors for opioid/SU with complementary cognitive-behavioral (change thoughts, beliefs, and behaviors) and relational approaches (improve trust, emotional regulation, appropriate parental response) [33–35]. Given that youth discharged from secure residential facilities often return to existing family/living environments, empowering the adults who are responsible for their care with effective tools and support is necessary and represents an urgent and potentially transformative prevention/intervention opportunity.

The purpose of the Leveraging Safe Adults (LeSA) Project is to examine the effectiveness of Trust-based Relational Intervention® (T﻿BRI) [36, 37] in preventing opioid and other SU among youth after release from secure residential facilities. The primary aim is to leverage existing relationships to more effectively support youth after returning home. Caregivers are trained to be “safe adults” for their youth, by building trust, practicing authentic communication, developing boundaries, and setting realistic expectations in order to proactively and effectively identify and address their youth’s needs. Through their relationships with safe adults, youth learn and practice self-regulation, enabling them to more effectively refrain from opioid use, other SU, and other risky activities.

**Fig. 1:**
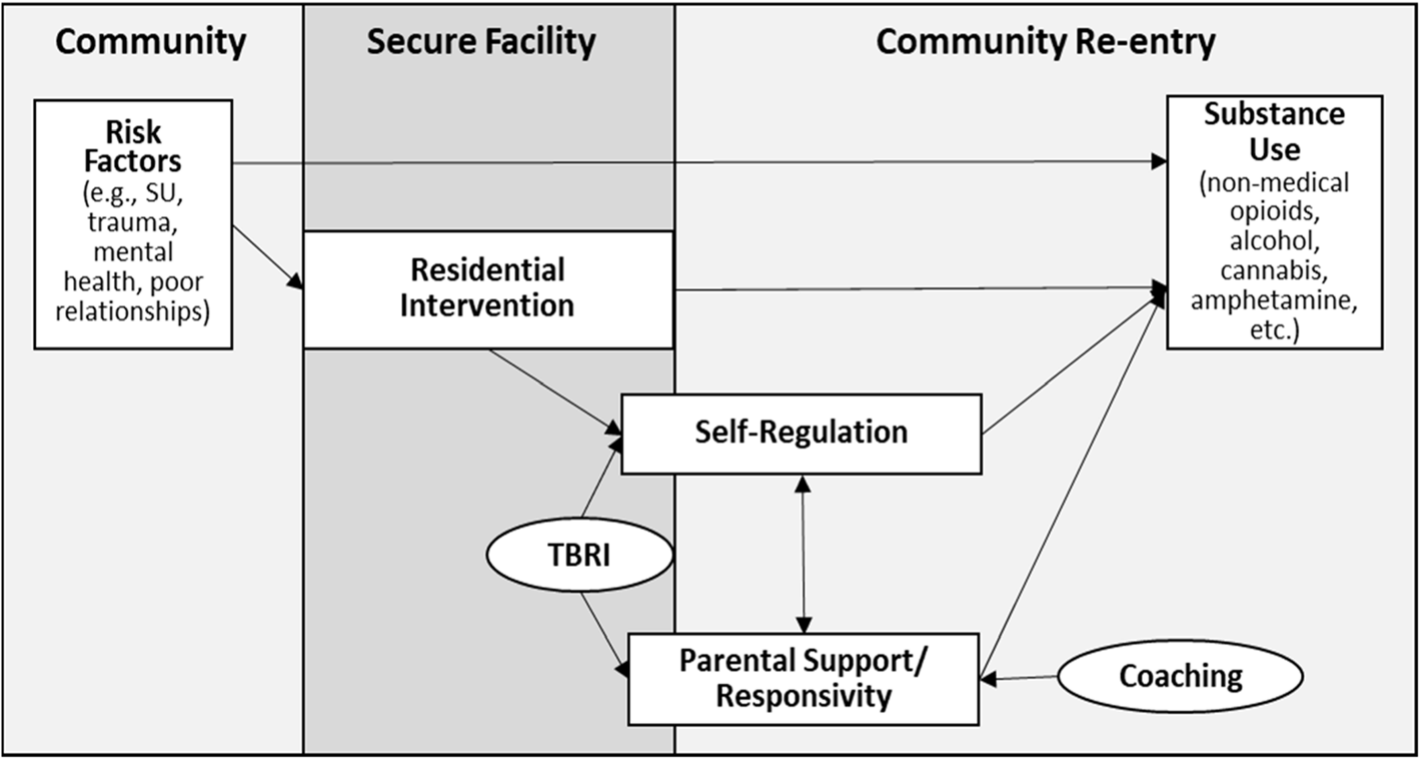
LeSA Project Conceptual Framework. *Note*. TBRI® = Trust-based Relational Intervention®

As depicted in Fig. 1, youth enter the JJ system (and subsequent secure residential settings) with significant risk for SU. TBRI is designed to strengthen relationships by improving the connection between youth and caregiver, empowering caregivers to identify and address youths’ physiological and emotional needs, and equipping caregivers with effective tools for correcting inappropriate behavior while maintaining healthy connection [36, 38]. By simultaneously addressing the underlying pillars of trauma-informed care (e.g., connection, self-regulation) [39, 40], caregivers learn effective support and response strategies, and youth learn to develop healthy relationships and practice self-regulation in an emotionally safe environment. This in turn strengthens trust and feelings of emotional safety, which promotes better decision-making and healthy lifestyle choices (including lower SU).

Because a high proportion of JJ youth has complicated living situations (e.g., child welfare involvement after allegations of abuse or neglect) [41], the term “safe adult” includes guardians (e.g., grandparent, aunt/uncle) as well as biological, adoptive, and foster parents. Safe adults (to whom youth will likely be released, determined by the presiding Judge and/or court system) agree to support the youth’s transition back to the community by providing housing and carrying out post-release plans (e.g., encouraging engagement in school and/or employment; helping navigate social and emotional challenges; transporting them to appointments). If successful, this project will provide a family-centered intervention for adolescents that can be implemented by JJ agencies and/or other social service agencies that intersect with JJ as part of re-entry services.

## Methods/design

### Research aims

The specific aims of the LeSA Project are to: (1) test the effectiveness of the TBRI intervention and its different support formats – no in-home TBRI support, Structured Coaching, Responsive Coaching – on (a) preventing initiation and/or escalation of opioid use, (b) public health and public safety outcomes, (c) putative change mechanisms, including self-regulation, psychosocial functioning, and relationship with a safe adult; (2) document factors related to TBRI implementation and sustainment; and (3) estimate the start-up and implementation costs and cost-effectiveness of TBRI support options relative to standard re-entry practices in achieving lower rates of opioid initiation and reductions in healthcare and JJ system costs.

Primary hypotheses are as follows:

- H1: Compared to youth receiving Standard Re-entry Practices (SRP) only, youth receiving TBRI in addition to SRP are less likely to initiate opioid use following discharge.
- H2: Compared to no in-home TBRI support, youth receiving Structured or Responsive Coaching are less likely to initiate opioid use following discharge, with the longest time to initiation occurring among youth receiving Responsive Coaching.
- H3: TBRI is most effective for preventing opioid initiation when facilities routinely use TBRI strategies within their residential settings, given increases in continuity as the youth transitions home from residential care.
- H4: The cost of implementing TBRI + SRP is higher than SRP alone, with higher cost-effectiveness when TBRI is added.

### Trust-based Relational Intervention®﻿

TBRI ﻿is a whole-child, attachment-based, trauma-informed intervention that promotes emotional regulation through interaction with responsive adults. The model is grounded in attachment theory and research [42, 43], emphasizing mindful awareness and engagement between adults and children as a means of building trust and resilience [37]. TBRI is also designed to address relational trauma, for instance, in the three pillars of trauma-wise care: felt-safety, connection, and self-regulation [39, 40]. The three evidence-based principles of TBRI include connection, empowerment, and correction [37]. Each principle includes a set of specific strategies that address social, behavioral, emotional, physiological, cognitive, and ecological aspects of children’s development and well-being (see Table 1).

**Table 1:** TBRI Principles and Strategies

TBRI Principle	TBRI Strategies	Examples
Connection (relationships and attachment needs)	Engagement	Appropriate touch, eye contact, playful interaction, behavior matching or mirroring
	Mindfulness	Empathy, receptive communication, physical presence, removing fears, felt safety, awareness
Empowerment (physical needs)	Physiological	Nutrition, hydration, sensory needs or factors, snacks, sleep and rest, physical activity
	Ecological	Rituals, schedules, transitions (e.g., creating transitional plan for youth re-entry), artifacts (e.g., “brain” hats, coloring workbooks)
Correction (behavioral needs; disarm fear-based behaviors and teach appropriate social skills)	Proactive	Life Value Terms (with respect, using words, gentle and kind), behavioral scripts, choices, compromises, “redos”
	Responsive	IDEAL Response (i.e., correction needs to be immediate, direct, efficient, action-based, leveled at behavior), Levels of Response (i.e., redirect using the least corrective effort, from playful, structured, calming, to protective engagement; “keep it low”)

*Note*. Please see Purvis (Purvis et al., 2013) for a detailed description of these TBRI strategies

The *primary TBRI intervention* is delivered while youth are in secure residential settings, within 3 months prior to discharge. Principles and strategies are learned and practiced in separate caregiver and youth group sessions, then strengthened in joint youth-caregiver sessions. The focus of caregiver sessions is on identifying and meeting youth needs, supporting appropriate self-expression, improving self-regulation, and developing healthier relationships. Youth are also trained in complementary sessions to develop skills and a deeper understanding of felt safety and trust. Key constructs are taught through playful engagement and role play for youth and caregivers to actively connect with one another while developing new skills.

The *primary TBRI intervention* is an adapted version of the TBRI Caregiver Curriculum [33, 37] and includes several components (see Tables 2-3): (1) Youth Trainings: 9, 45-min youth-only modules; (2) Caregiver Trainings: a 1-h individual introductory module and 9, 90-min caregiver-only modules; and (3) Nurture Groups: Youth and caregivers participate together in 4, 1-h joint-roleplay sessions. Both Youth and Caregiver Trainings are designed to be delivered in a group setting with three to five in each group; however, missed sessions can be delivered as individual sessions. Additional monthly “booster” nurture group sessions are delivered in instances where the youth’s discharge is extended beyond the original expected date.

**Table 2:** A Brief Overview of TBRI Curriculum (Caregiver Trainings, Youth Trainings, Nurture Groups)

Primary TBRI Intervention			
**Module**	**Youth and Young Adult Curriculum **	**Caregiver Curriculum**	**Nurture Group Activity (joint roleplay by youth/caregivers)**
0	• Introductions and Expectations • Familiarization with the Virtual Platform • Overview of the Tools for Learning and the Youth Workbook • Group rules: Designed for Felt Safety among all Participants	• Introductions and Expectations • Familiarization with the Virtual Platform •Overview of the Tools for Learning and the Caregiver workbook • Group rules: Designed for Felt Safety among all Participants	• N/A
*Session 1: TBRI: Introduction and Overview*			
1	• Explanation of a whole-person approach • Building relationships grounded in connection. • Overview of TBRI Principles	• Understanding TBRI, Risk Factors and Brain Growth • Overview of TBRI Principles	• Divided Line Drawing: Current versus future family • Begin communicating/ negotiating changes needed
2	• Balancing Structure and Nurture • Caregiving Styles • Preparation for TBRI Nurture Groups	• Balancing Structure and Nurture • Caregiving Styles • Preparation for TBRI Nurture Groups	
*Session 2: Connecting Principles*			
3	• Attachment • Four Hallmarks of a Secure Adult • Rupture and Repair • The Importance of an Apology	• TBRI Connecting Principles • Attachment	• Role Play: Apologizing • Modeling connecting after a rupture in the relationship
4	• Connecting • Mindfulness • Emotional Safety	• Mindfulness • Adult Attachment • TBRI Engagement Strategies	
*Session 3: Empowering Principles and Correcting Principles: Proactive Strategies*			
5	• Understanding the components of a Nurture Group	• TBRI Empowering Principles • Physiological Strategies • Sensory input and needs • Ecological Strategies	• Creating a Family Life Value Terms List • Communicating about strategies family will use upon the youth’s transition home
6	• Balancing Structure and Nurture • Practicing supportive communication	• TBRI Correcting Principles: Proactive Strategies • Choices, Compromises, Sharing Power • Life Value Terms	
*Session 4: Correcting Principles: Responsive Strategies*			
7	• Ecological Strategies • Three goals of correction: connection, contentedness, and changed behaviors	• Review of Correcting Principles: Proactive Strategies • The IDEAL© Response	• Role Play: Supportive vs. aggressive communication style • Engage in conversation about changes in family dynamics
8	• Power Struggles and Sharing Power • Life Value Terms • Role Play-IDEAL© Response • Graduation	• Levels of Response© • Applications using case studies • Graduation	

*Note*. The overall goals of this program include developing a spoken TBRI language and understanding for the youth to relate to their caregiver. They will be given voice, gain an understanding of self-awareness, develop autonomy, and most importantly, felt safety. This program is deeply rooted in connection, mindfulness, and trust

**Table 3:** A Brief Overview of TBRI In-Home Training Curriculum

Secondary TBRI Intervention		
**Module**	**In-Home Coaching Curriculum**	**Support Formats**
*Goals: (1) Creating an environment of felt-safety between coach and family and encourage felt-safety between family members (2); Facilitating a discussion about healthy relationships that meet the needs of all family members; and (3) Practicing strategies for engagement and mindful awareness.*		
1	• Felt Safety • Felt-safety in relationships • Strategies for building felt-safety	• *Structured Coaching: Required* • *Responsive Coaching: Required*
2	• Building Healthy Relationships • 4 Skills of Healthy Relationships • TBRI Connecting Strategies	
*Goals: (1) Creating space for an ongoing dialog about balancing structure and nurture in the caregiver-youth relationship (2); Practicing looking for the need behind the behavior (3); Engaging caregivers and youth in considering what shared power looks like in their family; and (4) Practicing setting the bar for growth and success.*		
3	• Balancing Structure and Nurture • Rethinking discipline • The meaning behind the behavior • Negotiating needs	• *Structured Coaching: Required* • *Responsive Coaching: Optional*
4	• Sharing Power • Obstacles to sharing power • Self-worth & autonomy • Appropriate expectations	
*Goals: (1) Discussing how stress changes our brain, and (2) Discussing using brain development as a lens to view adolescence.*		
5	• Toxic Stress and the Brain • The teen brain • Adverse Childhood Experiences	• *Structured Coaching: N/A* • *Responsive Coaching: Optional*
*Goals: (1) Returning to the topic of healthy relationships, and (2) Engaging the family in a deeper dive of how relationships shape lives.*		
6	• Adult Attachment • Experiences in close relationships • Earned security	• *Structured Coaching: N/A* • *Responsive Coaching: Optional*
*Goal: Building mindfulness practices to increase awareness of self, others, and situation*		
7	• Mindfulness • Grounding/breathing techniques • Self-care	• *Structured Coaching: N/A* • *Responsive Coaching: Optional*
*Goal: Discussing sensory processing with a focus on identifying and meeting specific sensory needs*		
8	• Sensory Experiences • Sensory profile self-assessment • Sensory resources	• *Structured Coaching: N/A* • *Responsive Coaching: Optional*
*Goals: (1) Creating space for discussing challenges around the family’s current transitions, and (2) Anticipating future transitions*		
9	• Life Transitions • Predictability and uncertainty • Detective diaries	• *Structured Coaching: N/A* • *Responsive Coaching: Optional*

*Note*. Sessions can be repeated with families who choose to continue coaching over a longer period of time (responsive coaching condition only)

The *secondary TBRI intervention* is provided after completion of the primary intervention and includes in-home support following discharge. Youth and caregivers randomly assigned to Coaching receive either Structured (4 TBRI In-Home Trainings) or Responsive Coaching (2+ TBRI In-Home Trainings with additional trainings as requested by the family or triggered by SU or risk factors observed/identified by research staff).

For the purpose of this project, TBRI intervention components are delivered by 1–2 Research Assistants (RAs) who are also trained as TBRI Practitioners. All TBRI Practitioners have a minimum Bachelor’s Degree in human services areas, more than 2 years of clinical experience and fieldwork in high-risk juvenile and adult populations, receive a minimum of 20-h onboard training and skills development as well as ongoing training/supervision. All caregiver sessions are delivered virtually to enable caregivers who do not live close to the site where their youth resides. Sessions are synchronous, and participants are provided an “orientation” to the online platform prior to the first session. Youth sessions are delivered in person at the facility by RAs, with support from non-correctional staff (e.g., clinical staff). Nurture groups are delivered in a group format with youth joining virtually as a group from the facility and caregivers joining individually from their homes. Sites determine when and where youth sessions will be delivered, including which staff to involve (i.e., therapeutic staff who could participate in intervention activities. Staff are provided training on logistics and strategies to address potential behavioral issues and support a youth in need.

In addition to the features described above, TBRI also serves as a multi-systemic caregiving model that has been successfully implemented in a wide variety of professional contexts [44] [45] [46] [47]. TBRI can be implemented by anyone trained in the tools and strategies, and therefore provides a framework and impetus for empowering all adults, regardless of status or education, to be catalysts of change for youth. Therefore, TBRI can be implemented across an entire JJ system to facilitate the use of trauma-informed approaches in all interactions. For the purpose of this project, the implementation of TBRI principles and strategies within the broader residential context is referred to as the *organizational TBRI intervention*.

### Overall study design

The LeSA Project uses an effectiveness/implementation Hybrid Type 1 Design [48] to simultaneously test the effectiveness of TBRI for preventing non-medical use of opioids among JJ-involved adolescents and gain insight into facility-level obstacles that must be overcome if the intervention is to be implemented and sustained as part of JJ re-entry protocols. The overall study design includes (a) a delayed-start at the site level and (b) a randomized controlled trial at the participant level.

To address the logistical burden of protocol implementation and to control for the influence of other exogenous factors [49], participating sites are assigned to one of three Study Waves and proceed through three sequential study stages accordingly. Wave membership is determined by stratifying by state (3 sites in one, 6 in the other) and ranking by the number of youth served in descending order. For the state with 3 sites, one is assigned to each wave, with the highest populated site starting in Wave 1. For the state with 6 sites, two are assigned to each wave, with the highest and fourth-highest populated sites starting in Wave 1. Stage 1 - SRP data collection launches as soon as sites are ready. Stage 2 study activities are delayed by 1 month.

### Hybrid design: effectiveness trial

The effectiveness trial includes a comparison of youth enrolled in SRP to youth enrolled in SRP plus TBRI, whereby each facility serves as its own control. Because caregivers may need support after their youths’ release, three versions of TBRI are compared, with youth in the SRP + TBRI cohort being randomly assigned to one of three support formats: (1) TBRI Training only (no in-home TBRI support), (2) TBRI Training + Structured Coaching (4 sessions), and (3) TBRI Training + Responsive Coaching (2+ sessions, triggered by youth need/risk).

**Fig. 2:**
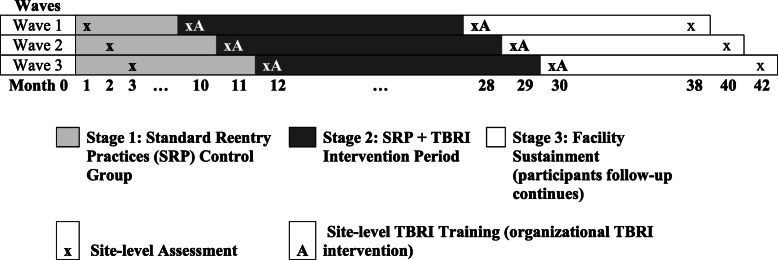
Recruitment and data collection timeline

The effectiveness trial proceeds in 3 stages: Standard Reentry Practice (SRP) only (baseline control), SRP + TBRI, and Facility Sustainment (see Fig. 2). **Stage 1 - SRP**: sites engage in standard practice; study activities include youth and caregiver assessments over 18 months. Each site serves as its own control. A total of 120 youth/adult dyads are recruited across the nine sites. The length of SRP varies between 8 months (Wave 1) and 12 months (Wave 3). **Stage 2 - SRP + TBRI Intervention:** sites continue to engage in standard practice; TBRI intervention is delivered to youth and caregivers enrolled in the study; youth and caregiver assessments are conducted over an 18-month period. A total of 240 youth/adult dyads are recruited across the nine sites. **Stage 3 - Facility Sustainment**: youth/caregiver recruitment, baseline assessment, and interventions cease; youth and caregiver follow-up interviews continue; responsibility for implementing primary and secondary TBRI intervention components is transferred to the JJ facility.

**Fig. 3:**
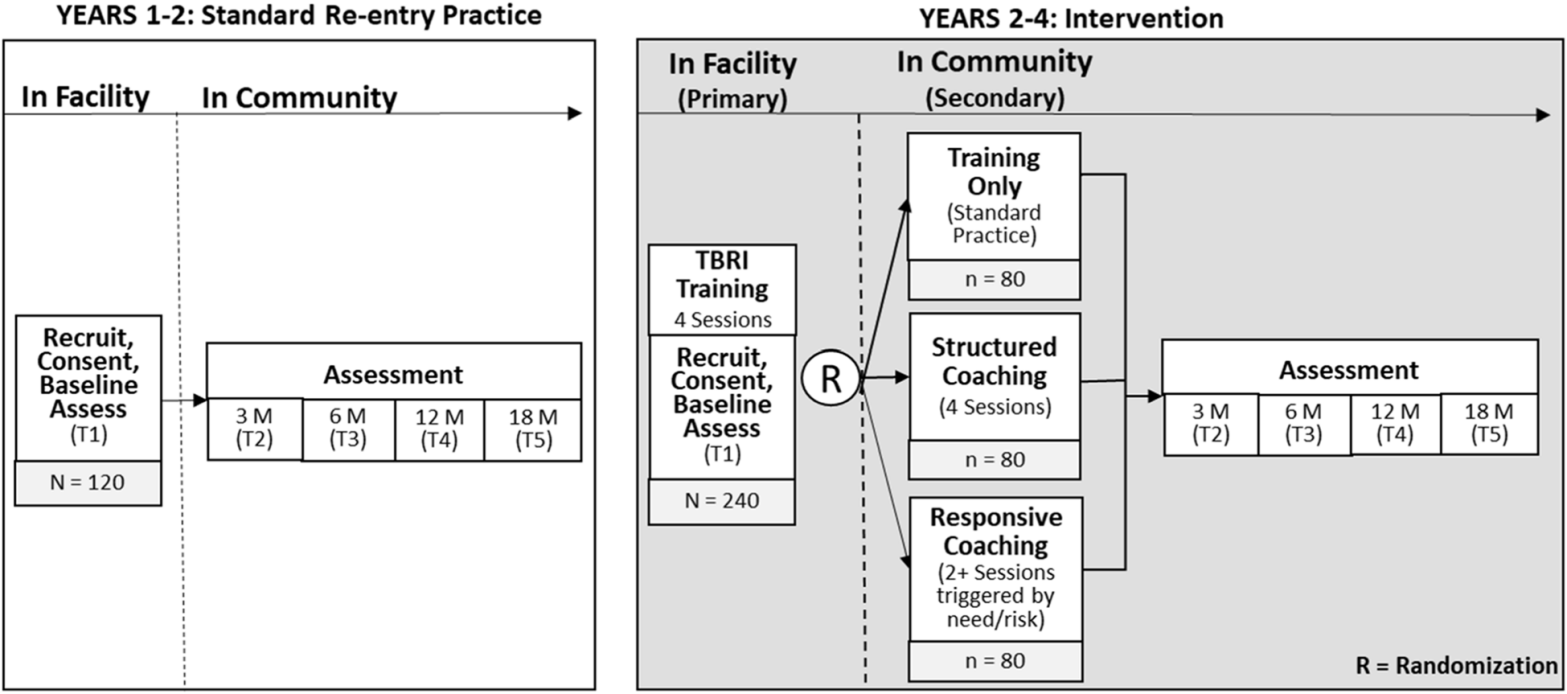
**Research design: Randomized control trial (*N*** **= 360 dyads).** *Note*. TBRI In-Home Training: either Structured Coaching or Responsive Coaching; Virtual or physical in-home visits by a TBRI Practitioner. Responsive Coaching “trigger” would be (**a**) substance use (SU), (**b**) parent suspects SU, (**c**) presence of risk factors for SU

At the participant level (see Fig. 3), for both SRP and SRP + TBRI stages, youth and safe adults are recruited/consented 2–3 months prior to discharge, and individually complete baseline at recruitment and follow-up interviews at 3- (within 2 weeks of discharge), 6-, 12-, and 18-months post-baseline. For dyads recruited during the Stage 2 – SRP + TBRI Intervention period, the primary TBRI intervention sessions begin after baseline assessment while the youth are in the residential facility. At the point of discharge, dyads are randomly assigned to one of 3 TBRI in-home support conditions, with probabilities equal to 1:3 within each site. This overall design enables comparison of TBRI versus SRP plus a randomized control trial comparing TBRI support conditions.

#### Randomization

Dyads are randomly assigned to TBRI support condition by the research project director using urn randomization until the recruitment target is met (minimum of 80 per arm; an average of nine per arm, per site). Urn randomization is used to balance the characteristics of youth by group on sex (male vs. female), race (Caucasian vs. other race), and primary speaking language (English vs. Spanish).

### Hybrid design: implementation overlay

The implementation overlay includes three types of research activities occurring throughout all three project stages: (1) documenting facility use of trauma-informed strategies and contextual factors (see *Measurement and outcomes*) that facilitate or impede the use of these strategies, (2) supporting facility efforts to become more trauma-informed and in their use of TBRI practices during daily interactions with youth (see *Support for the organizational TBRI intervention*), and (3) building the necessary infrastructure to sustain the primary and secondary TBRI intervention components after the project ends (see *Sustaining the primary and secondary TBRI intervention*).

Prior to the launch of Stage 1 - SRP, the research team and facilities work together to explore each facility’s needs and desire to implement trauma-informed strategies such as TBRI. Contextual factors and use of trauma-informed strategies are documented through surveys of JJ facility staff at four time points throughout the study: the beginning of Stages 1, 2, and 3, and the end of Stage 3 (see Fig. 2). This information is used to customize support for facilities seeking to implement TBRI across their system and to understand how contextual factors—specifically how the use of trauma-informed care at a facility level—moderate the effectiveness of the primary and secondary TBRI intervention.

#### Support for the organizational TBRI intervention

While the project focuses on the delivery of the primary and secondary TBRI intervention with youth and caregivers, all facilities are invited but not required to participate in the organizational TBRI intervention. All facilities receive two 1-day TBRI trainings as part of the LeSA project at the beginning of Stages 2 and 3 (see Fig. 2). Training focuses on basic constructs underlying TBRI and an introduction to TBRI principles and strategies. For facilities that elect to implement TBRI across their residential program and/or across their juvenile systems, additional support is provided by the research team. An important step is to develop a change team within each facility, ideally with representation from every level, including JJ leadership (e.g., Deputy Director), behavioral health staff (e.g., clinicians), and other staff with youth interaction (e.g., direct care, supervising officers). This group is responsible for leading TBRI implementation efforts within their system.

Facilities can participate in two types of activities to cultivate TBRI expertise: (1) coaching and support for how to adapt TBRI principles and strategies within their system and (2) TBRI Practitioner training for 2–4 facility staff to further support and sustain the implementation of TBRI. Using a train-the-trainer approach, these TBRI Practitioners then extend TBRI principles and strategy use across departments and levels of the system, demonstrating strategies, training staff, and providing internal expertise on ways to modify existing practices. These opportunities are provided at any point during the project.

#### Sustaining the primary and secondary TBRI intervention

Stage 3 – Facility Sustainment, affords a naturalistic investigation of sustainment of the intervention within the facilities after responsibility for intervention delivery has been transferred from the research center to the JJ facilities. Prior to the end of Stage 2 – SRP + TBRI, sites are asked to indicate whether they would like to continue offering the primary and secondary TBRI intervention after the effectiveness trial ends. Sites that choose to do so collaborate with the research team to develop a customized plan for transitioning intervention delivery to facility staff, using information collected as part of the effectiveness trial. Facilities can choose how best to implement the curricula so that it fits within their existing policy, practice, staffing, and financial constraints. Research staff provide training in all aspects of curriculum delivery and support initial efforts as needed. Specific details regarding facility implementation plans and inner/outer contextual factors that impact decisions will be documented [50].

### Study samples and recruitment

A total of nine residential facilities designed to address the behavioral and emotional needs of justice-involved youth serve as research sites for this project. Three are located in a Midwestern state and 6 in a Southwestern state in the U.S.A. Three are operated by the state JJ system, 4 by local JJ departments, and 2 by an independent agency. All sites are secure residential programs with medium security protocols, all receive referrals from JJ departments across a wide geographic region, and all provide educational, medical, and behavioral health services. At project start, 3 sites had incorporated TBRI into their trauma-informed care approach for 1–3 years, 2 were familiar with TBRI but not using principles and strategies, and 4 were unfamiliar with TBRI.

The project involves two target samples drawn from the nine facilities at rates proportional to current census/staffing levels. The effectiveness trial sample includes a total of 360 youth/caregiver (e.g., parent/guardian, extended family member) dyads recruited from participating JJ facilities over a 3-year period (approximately 10–15 dyads/year per site). Inclusion criteria include youth ages 15–18 at study enrollment who (a) are disposed to community supervision (i.e., probation) and receiving care in a secure JJ facility, (b) have no indication of active suicide risk, and (c) have worked with JJ staff to identify one caregiver that is willing to participate in the study. The implementation overlay sample includes approximately 203 (out of 810 potential participants with a 25% completion rate) JJ staff (facility staff and probation officers; an average of 22–23 per site) who work directly with youth during residential and/or community re-entry. Out of this sample, approximately 90 JJ staff (an average of 10 per site) are recruited for focus groups.

Research sites facilitate the identification and recruitment of family participants (e.g., connecting RAs to families). Brochures, consent and assent forms, assessment battery, and curricula are available in English and Spanish. Bilingual English and Spanish-speaking RAs are available for Spanish-speaking families in all research activities. The research protocol is approved by the Texas Christian University (TCU) Institutional Review Board (IRB) and by site research review committees (if applicable), and the local juvenile board (if applicable). Assessment data are collected via Qualtrics. Protocols are delivered via secure virtual platforms (e.g., Zoom, Microsoft Teams) or in-person, depending on the activity. Caregiver consent, parental/guardian permission for youth participation, and youth assent are obtained in writing by RAs prior to all research activities; youth provide consent in writing if they turn 18 during the study. Youth and caregivers are compensated separately for their time spent in assessments in an amount of $15 e-cards per assessment.

Research staff work with JJ facility leadership to determine participant lists for staff surveys and focus groups. All individuals with direct youth contact (e.g., case managers, educators, direct care staff, probation officers) receive an individualized link to a confidential survey administered via Qualtrics. Recruitment of newly hired staff occurs prior to each assessment. JJ leadership are asked to identify up to 10 individuals representing leadership and line staff, including admin, clinical supervisors, justice probation officers, clinical staff, direct care staff, for in-person focus groups. To avoid potential coercion, introduction of risk to subordinates’ disclosures, and/or biased/invalid focus group data, leadership and line staff are invited for separate in-person focus groups at each site. Consent is obtained prior to the first assessment by an RA and prior to each focus group interview. Staff participants receive an honorarium (e.g., a cup, group meal) for participation.

### Measurement and outcomes

For the effectiveness trial, data come from 3 sources: (1) youth self-report at Months 0, 3, 6, 12, and 18; monthly post-release check-ins, (2) JJ facility records (e.g., recidivism, treatment utilization), and (3) caregiver self-report with a timeframe identical to youth. Table 4 depicts research goals, assessment instruments, data collection timeline, and youth and/or caregiver single or dyadic perspective on each construct. The primary outcome is *days to youth initiation* of non-medical opioid use: measured by (a) Timeline Followback [51], (b) TCU Drug Screen 5 with Opioid Supplement (TCU DS 5-OS) [52], and (c) Substance Use Involvement items from the HEAL Prevention Cooperative [53]. Secondary outcomes include putative change mechanisms, public health, and public safety outcomes. Putative change mechanisms include youth self-regulation and self-efficacy (e.g., “How confident are you that you will not misuse a prescription opioid in the next 30 days”) [53] and youth/caregiver psychosocial functioning and relationships between youth and caregivers. Self-regulation is measured with the TCU Adolescent Thinking forms on regulation (TCU THK-NUY, TCU THK-PUY; decisions influenced by positive or negative emotion) [54], supplemented with the delayed discounting task (DD task) [55], Difficulties in Emotion Regulation Scale (DERS) [56], and Barkley Deficits in Executive Functioning Scale-CA Short Form (BDEFS-CA) [57]. *Psychosocial functioning* is measured by the Strengths and Difficulties Questionnaire for youth (SDQ) [58], Generalized Anxiety Disorder Assessment (GAD-7) [59], Patient Health Questionnaire (PHQ-8) [60], PROMIS-Pain [61], and items that evaluate youth’s self-reported efficacy to resist opioids (“How confident are you that you will not misuse a prescription opioid in the next 30 days?”) [53]. *Youth/caregiver relationships* are measured using the Family Assessment Device (FAD) [62], the Experiences in Close Relationships – Relationship Structures (ECR-RS) [63], and the Attachment, Relational Frustration, and Discipline Practices scales from the Behavior Assessment System for Children Parent Relational Questionnaire (BASC-3) [64].

**Table 4:** An overview of assessment and timeline for the effectiveness of TBRI intervention

Construct and Instrument	Measurement Time Points						Participant	
	Baseline	MO 3	MO 6	MO 12	MO 18	Monthly check-in	Youth	Caregiver
Youth SU								
TLFB		x	x	x	x		x	
TCU DS 5 - OS	x	x	x	x	x		x	
HEAL SU Involvement	x	x	x	x	x		x	
Monthly check-in						x	x	
JJ records	Administrative records (Years 2–5)						NA	NA
Youth Self-regulation								
TCU THK, NUY and PUY	x		x	x			x	
DD task	x	x		x			x	
DERS	x	x	x	x	x		x	
BDEFS-CA	x		x	x				x
SDQ	x		x		x		x	x
GAD	x	x	x	x	x		x	x
PHQ-8	x	x	x	x	x		x	x
PROMIS Pain	x	x	x	x	x		x	x
Self-efficacy	x	x	x	x			x	
Monthly check-in						x	x	
Youth/Safe Adult Relationship								
FAD	x	x	x		x		x	x
ECR– RS	x	x		x	x		x	x
BASC-3	x		x	x				x
Monthly check-in						x	x	
Public Health and Safety								
Opioid-related health	x	x	x	x	x		x	x
JJ records	Administrative records (Years 2–5)						NA	NA
Family Background								
Background	x						x	x
TCU DS 5- OS	x							x
ACEs	x						x	x
Social SU exposure	x	x	x	x	x		x	
Parenting								
PMI	x	x						x
CGSQ-SF	x	x	x					x
RS	x							x
Service utilization		x	x	x	x		x	x
COVID-19 impact	x	x	x	x	x		x	x
Utilization of TBRI Principles								
Utilization and Importance		x	x	x	x			x
CAWS	Administered at end of TBRI Group Training and In-Home Training							
Intervention fidelity	Administered at post-TBRI session						x	x

Note: TLFB = Timeline Followback, TCU DS 5-OS = TCU Drug Screen 5 with Opioid Supplement, HEAL SU Involvement = Substance Use Involvement items from the HEAL Prevention Cooperative, TCU THK- NUY and PUY = TCU Adolescent Thinking Forms-Negative and Positive Urgency Scales, DD task = Delayed Discounting Task, DERS = Difficulties in Emotion Regulation Scale, BDEFS-CA = Barkley Deficits in Executive Functioning Scale -CA Short Form, SDQ = Strengths and Difficulties Questionnaire, GAD-7 = Generalized Anxiety Disorder Assessment, PHQ-8 = Patient Health Questionnaire, Self-Efficacy = Self-Efficacy to Avoid Opioids, FAD = Family Assessment Device, ECR-RS = Experiences in Close Relationships – Relationship Structures, BASC-3 = Behavior Assessment System for Children- Parent Relational Questionnaire, ACEs = Adverse Childhood Experiences, PMI = Parent Motivation Inventory, CGSQ-SF = Caregiver Strain Questionnaire- Revised Short Form, RS = Resiliency of Self-Efficacy Scale, Utilization and Importance of TBRI = Perceived Importance and Use of TBRI Strategies, CAWS = Child and Adolescent Well-being Scale; at MO 3, only the Affective Responsiveness scale from the FAD will be administered; at each time point, only the Attachment, Relational Frustration, and Discipline Practices scales from the BASC-3 will be administered; for baseline the trait version of DERS will be administered and for all follow-ups the state version of DERS will be administered; CAWS and Intervention Fidelity will both be reported by TBRI Practitioners; Baseline = initial assessment, MO 3 = 3 month follow-up, MO 6 = 6 month follow-up, MO 12 = 12 month follow-up, MO 18 = 18 month follow-up

*Public health and safety outcomes* include opioid-related health, opioid use overdose, use of other illegal substances, recidivism, and SU or MH treatment records. *Opioid-related health* outcomes include SU treatment, hospital visits related to SU, diagnosis, overdose, and receipt of Narcan [65]. *Recidivism* information (re-arrest, re-adjudication), urinalysis results, and *treatment* records (type of referral, service receipt) come from JJ facility youth records [65]. For these data, sharing agreements between each state-level JJ facility and the research institute are established, specifying how de-identified records are shared, stored, and analyzed, following models from previous collaborations [66]. *Background information* on youth is extracted from existing JJ youth records (e.g., offense, disposition decision, supervision level, offense history including truancy, and prior SU or MH diagnosis).

Youth and caregivers provide different pieces of background information that serve as covariates or moderators, including demographics (e.g., age, sex, race/ethnicity, employment, education). Caregivers are also asked about their SU (TCU DS 5-OS) [52] and relationship with their own caregiver using the Experiences in Close Relationships-Relationship Structures (ECR-RS) [63]. Other potential covariates include number and type of ACEs [67], youth self-reported social exposure to substance use (e.g., “How often does the adult who is most important to you drink alcohol?”) [53], services youth and family received (adapted from the Child and Adolescent Services Assessment) [68], caregiver motivation in treatment participation (Parent Motivation Inventory; PMI) [69], parenting strain (Caregiver Strain Questionnaire-Revised Short Form; CGSQ-SF) [70], and parental resiliency (Resiliency of Self-Efficacy Scale; RS) [71]. Participants are also asked to respond to two open-ended questions regarding the impact of COVID-19 (e.g., “How is the COVID-19 crisis affecting you or has affected you?”). *Monthly phone check-ins* with youth assess 8 indicators of any opioid use; any alcohol, other drug use; truancy; trouble with the law, increases in depression, increases in anxiety, increases in the stress in relationship with the safe adult in the past month.

Participants in the SRP + TBRI cohort are asked to report on the utilization of intervention content and intervention fidelity, which are developed specifically for this project. Caregivers’ use of TBRI strategies is assessed by (1) self-report after completion of final TBRI Group Training session and after each TBRI In-Home Training session and (2) RA report upon completion of each TBRI Nurture Group and TBRI In-Home Training sessions. RAs and participants complete a post-session checklist after each session to assess fidelity of content delivery and participant engagement.

For the implementation assessment, the LeSA JJ staff survey (see Table 5 for a description and timeline) includes information on organizational context (Survey for Juvenile Justice Secure Facilities) [72], staff characteristics (Survey of Organizational Functioning and Leadership, Professional Quality of Life; SOFL, PQL) [73], use of trauma-informed care (Attitudes Related to Trauma-Informed Care Scale; ARTIC) [74], use of TBRI intervention strategies (TBRI Acceptability, Appropriateness, and Feasibility scale), and TBRI Professional Use scale [75], and answer two open-ended questions regarding the impact of COVID-19. Youth participating in LeSA also report their perceived social support from facility staff (with the Berlin Social Support Scale; BSSS) [76]. During focus groups, staff report routine practices in the use of trauma-informed approaches (e.g., “How does your facility address issues that youth may have had due to their traumatic experience(s)?”) and child-centered strategies (e.g., “In what ways does your agency strive to meet the unique needs of youth?”), agency facilitators/barriers to uptake of new interventions (e.g., “What (if any) changes have you made that produced significant changes for youth?”), general penetration of TBRI within the agency (e.g., “What (if any) changes have you made using TBRI that produced significant changes for the staff or your organization more broadly?”), and agency interest/success in sustaining and/or expanding TBRI practices (e.g., “Are you planning to continue TBRI practices at your agency?”). The focus groups will be held once per year over 4 years for approximately 60 min.

**Table 5:** An overview of assessment and timeline for the implementation

Construct and Instrument	Brief Description	Measurement Time Points				Participant
		Start of Baseline or New Recruitment	Start of Staff TBRI Training	End of Staff TBRI Training or Start of Follow-up	End of Follow-up	
Survey for Juvenile Justice Secure Facilities	Agency and youth characteristics, staffing, clinical assessment, behavioral, treatment services, family engagement.	x	x	x	x	Agency administrator or designated representative
Staff Background	Staff age, race, ethnicity, education, work experiences.	x				All JJ staff
SOFL, PQL	Climate and staff characteristics.	x	x	x	x	All JJ staff
ACEs	Staff ACEs	x				All JJ staff
ARTIC	Attitudes toward trauma-informed care.	x	x	x	x	All JJ staff
TBRI Acceptability, Appropriateness, and Feasibility	Staff attitudes toward TBR		x	x	x	All JJ staff
TBRI Professional Use	Staff use of TBRI strategies.	x	x	x	x	All JJ staff
COVID-19 Impact	COVID-19 impact on staff, work environment, and services for youth.	x	x	x	x	All JJ staff
JJ Staff Focus Group	Use of trauma-informed strategies, facilitators and barriers to uptake of new interventions, use of TBRI, agency interest/success in sustaining and/or expanding TBRI practices.	x	x	x	x	Leadership and line staff, separately.

Note: SOFL = Survey of Organizational Functioning, PQL = Professional Quality of Life, ACEs = Adverse Childhood Experiences, ARTIC = Attitudes Related to Trauma-Informed Care, BSSS = Berlin Social Support Scale

For economic analyses, the Drug Abuse Treatment Cost Analysis Program (DATCAP) [77, 78] is used to capture implementation and operating costs of TBRI [79] [78], generating cost summary statistics using data on intervention engagement and youth case flow (e.g., the average annual cost per youth; average cost per treatment episode per youth) [80] [81].

#### Power

For H1 examining the effectiveness of SRP + TBRI compared to SRP alone, the proposed Intent to Treat (ITT) sample will include 360 youth/safe adult dyads (120 SRP, 240 TBRI), with 80 randomized to each TBRI condition. SAS PROC POWER for rank tests comparing 2 survival curves found that, if overall opioid initiation rates are 35% (SRP) vs. 25% (all TBRI groups) at any assessed time point, power is estimated to be at 93% (*α* = .05). For H2 comparing the 3 TBRI conditions, SAS PROC POWER for rank tests comparing 2 survival curves found that for sample sizes of 80 in each group, power = .80 when the hazard ratio = 1.57. When the combined TBRI groups are tested against SRP, power = .80 when the hazard ratio = 1.37.

For H3 and H4 pertinent to the organizational TBRI intervention, the expected sample (nine sites, up to 90 staff participants per site) and the response rate of 25% gives an expected sample of 203 participants. A small effect size (0.20), a power of 0.80, significance level of 0.05, and an intraclass correlation of 0.10 are used to calculate the minimum required sample size. Repeated measures design comparing staff participants over time results in a minimum sample size of 43 participants. To be more conservative, the same parameters are used for a mixed-effect design (four repeated measures by nine sites), yielding a minimum sample size of 189 participants.

### Data management

#### Confidentiality

Participants are assigned study identification numbers to link data across times. Confidentiality of individual responses is enhanced by removing additional information connecting to personal identities during data cleaning if present. Staff participants are instructed to avoid using names throughout focus group sessions. All electronic records of online consent are kept in a secure server maintained by research staff at the research institute and are kept separately from the data. Electronic data are encrypted and password protected. To minimize the risk that youth, caregivers, or staff could potentially be individually identified by an outside entity, data are not released to individuals not affiliated with the project, which is consistent with the CFR Protection of Human Subjects and related protections and regulations. Researchers with access to data are required to sign confidentiality statements. Any published reports by research staff only include aggregated data, so no individual person can be identified. All data use and access are governed by these regulations, policies, and procedures.

#### Data sharing

The current protocol is part of the Helping to End Addiction Long-term Prevention Initiative (NIH HEAL Prevention Initiative) with a total of 10 research institutes and 1 Coordinating Center [82]. All data are further de-identified (e.g., by systematically scrambling Study IDs within study sites) before being shared with the HEAL Prevention Coordinating Center. The Coordinating Center does not have access to identifying information, nor do they have access to or knowledge of how participant study identification numbers are scrambled. At project close, data will be shared publicly, following the HEAL Prevention Guidance for Appropriate Public Access and Data Sharing Plans. Sharing of underlying primary data for the publications will be made broadly available through an appropriate data repository, such as the NIH HEAL Initiative central data repository, or a non-NIH repository that conforms to the principles articulated in the HEAL Public Access and Data Sharing Policy [83].

#### Data safety and monitoring

The LeSA Project activities are governed by a Data Safety and Monitoring Board (DSMB) established by the funding agency and comprised of prevention scientists functioning independently of the funding agency. The DSMB meets with the research team semi-annually to ensure the safety of participants and the validity and integrity of the data. Adverse events are monitored by the research team and reported to the University IRB within 72 h of notification and severe adverse events are reported to the IRB and funding agency within 24 h of notification.

### Data analysis plan

For all analyses, an ITT approach is used, where all study participants are included in analyses. For H1 and H2, comparisons of survival curves for each group are performed using SAS PROC PHREG and PROC GLIMMIX using the log rank test with the Sidak multiple-comparison adjustment, with the primary outcome as the dependent variable. Differences between groups on secondary outcomes and change over time in implementation measures (e.g., TBRI use) are examined using repeated measures ANOVAs (SAS 9.4, PROC MIXED), with study site as random effects and condition, time, and interactions as fixed factors. Because SU rates are generally lower among JJ females, sex as a biological variable is examined as a moderator of differences in outcomes using univariate analyses (e.g., chi square, t-tests, ANOVA); when significant, tests of moderation are undertaken as part of all analyses. Putative change mechanisms are investigated as mediators of the Group – Primary Outcome relationship and structural equation modeling (SEM; using Mplus) is used to examine sex differences in mediation. For H3, changes in the outcomes over time are examined using repeated measures ANOVAs (SAS 9.4, PROC MIXED), with study site as random effects and time and time × site interactions as fixed factors. For H1–3, a log rank test is used to assess study dropout; stochastic multiple-imputation provides maximum likelihood estimates, accounts for uncertainty in missing values, and provides unbiased estimates of the treatment effect if data are missing at random.

Qualitative data from focus groups are transcribed and qualitatively coded (in Atlas.ti) by at least 2 research staff. An iterative process of coding and thematic analysis [84] are used to understand routine practices employed, nuances in the use of trauma-informed and child-centered strategies, facility facilitators/barriers to uptake of new interventions, general penetration of TBRI within the facility, and facility interest in sustaining and/or expanding TBRI practices.

To address H4 regarding economic components, an activity-based micro-costing approach is used to identify, measure, and value resources invested by participating sites, and link costs to primary and secondary study outcomes [85]. Cost comparisons represent the incremental costs of adopting SRP + TBRI relative to SRP alone. Incremental cost represents the difference in facility/provider cost after adopting the intervention vs before adopting [86, 87], and incremental effectiveness is the difference in time to opioid use initiation in TBRI relative to SRP (and between TBRI support conditions). For each facility, differences in costs and incremental cost-effectiveness ratios are estimated using multi-level general linear models (e.g., generalized linear mixed models) to account for clustering at the facility/provider and patient levels. Nonparametric bootstrapping is used to compute confidence intervals [88].

## Discussion

The LeSA project addresses the opioid epidemic by focusing on justice-involved youth at risk for SU and substance use disorders (SUDs). As is well documented, most JJ-involved youth have experimented with using illegal substances and/or developed a SUD prior to entering the JJ system [66] [89] [19] [90] [27]. For this reason, and because youth most often return to the same homes and communities in which they formerly engaged in risky behaviors, the LeSA project is designed to empower existing caregivers—the adults to whom youth are released—with the ability to help prevent opioid and other drug use among JJ youth returning home after secure residential care.

In this project, TBRI is conceptualized as an “indicated” prevention intervention [31] aimed at addressing putative mechanisms underlying risky behavior and SU. Caregivers gain strategies to better support youth after release, strengthen youth-caregiver communication, enhance youth self-regulation, and proactively and effectively identify and address their youth’s needs. TBRI is not only a caregiver intervention but is also a trauma-informed caregiving model that can be applied in any caregiving setting. When the TBRI model is implemented by staff within JJ secure residential settings, strategies used by caregivers at home reinforce the youth’s experiences in the facility (e.g., how to calm themselves when they become dysregulated), resulting in greater continuity as youth transition from one caregiving environment to another. Consistency across multiple contexts within the youth’s broader ecosystem increases the likelihood that behavioral and emotional gains will be sustained [91] [92]. In this regard, the TBRI Caregiver Curriculum has the potential to supplement and extend gains made during residential care.

The Hybrid Type 1 effectiveness/implementation design enables a scientifically robust study of the effectiveness of TBRI while examining contextual factors that impact implementation. For example, using techniques such as multilevel modeling, the added value of TBRI (in addition to SRP) can be examined within various contexts (e.g., the degree to which TBRI and other trauma-informed strategies are used within the nine sites). The implementation component of the hybrid design focuses on developing internal JJ facility expertise to increase sustainment of the intervention and advances implementation science by piloting strategies for transferring responsibility for the intervention from the research team to facility staff at the project end.

While the project is designed to provide a robust test of TBRI within JJ contexts, potential limitations should be noted. Although representing diverse geographic locations and populations of youth, the nine participating facilities are drawn from only two states and may not be representative of all JJ facilities across the US. Furthermore, the sample of facilities is drawn from those with strong relationships with state leadership, rather than a randomly selected sample from a state-wide pool. The project launched during the COVID-19 global pandemic, and JJ facilities had to modify their admission, safety, and treatment protocols to address the health needs of youth and staff. While phase I activities began after the vaccine became available and health precautions lifted, some facility program elements remained affected, which could influence generalizability under non-pandemic circumstances. Furthermore, while the team successfully adapted in-person protocols to a synchronous virtual format, caution is needed if generalizing to fully in-person delivery formats.

If successful, the LeSA Project will result in important advances, including development of an effective attachment-based prevention intervention for high-risk adolescents and caregivers, strengthening the evidence base for TBRI, and contributing to the field of implementation science. With continuing efforts in improving service quality and practice, greater emphasis is placed on using prevention and treatment interventions that are considered “evidence-based practice” (EBP) [50]. TBRI is currently listed as a “promising practice” on the California Evidence-Based Clearinghouse for Child Welfare (CEBC) [93] and the Title IV- E Prevention Services Clearinghouse [94]. Data from the LeSA project has the potential to improve these ratings because the study design meets rigorous design and reporting requirements. Given that state funding is often tied directly to EBP ratings (e.g., reimbursement for the delivery of a well-supported EBP), interventions with higher ratings (e.g., supported or well-supported) are more likely to reach their intended audiences—families, and youth in need. Additional implications for implementation science include increased understanding of how to implement TBRI within complex systems such as JJ, how to transition responsibility for intervention from a developer to field-based staff at the end of a project, the costs (and cost-benefit) associated with delivering the intervention as part of routine practice, and what supports (e.g., logistical, training, coaching) are necessary to implement and sustain complex interventions that span multiple contexts and involve multiple components (e.g., in and out of the facility; staff and family members).

## Data Availability

See *Data Management* section.

## Abbreviations

ACEs: Adverse Childhood Experiences
ARTIC: Attitudes Related to Trauma-Informed Care
BASC-3: Behavior Assessment System for Children-Parent Relational Questionnaire
BDEFS-CA: Barkley Deficits in Executive Functioning Scale
BSSS: Berlin Social Support Scale
CAWS: Child and Adolescent Well-being Scale
CEBC: California Evidence-Based Clearinghouse for Child Welfare
CGSQ-SF: Caregiver Strain Questionnaire- Revised Short Form
DATCAP: Drug Abuse Treatment Cost Analysis Program
DD task: Delayed Discounting Task
DERS: Difficulties in Emotional Regulation Scale
DSMB: Data Safety and Monitoring Board
EBP: Evidence-based practice
ECR-RS: Experiences in Close Relationships-Relationship Structures
FAD: Family Assessment Device
GAD-7: Generalized Anxiety Disorder Assessment
HEAL: Helping End Addiction Long-term
IDEAL: Immediate, Direct, Efficient, Action- based, Leveled at behavior
IRB: Institutional Review Board
ITT: Intent to Treat
JJ: Juvenile justice
MH: Mental health
MO 3: 3-month follow-up
MO 6: 6-month follow-up
MO 12: 12-month follow-up
MO 18: 18-month follow-up
PHQ-8: Patient Health Questionnaire
PMI: Parent Motivation Inventory
PQL: Professional Quality of Life
RA: Research Assistant
RS: Resiliency of Self-Efficacy
SDQ: Strengths and Difficulties Questionnaire
SEM: Structural Equation Modeling
SOFL: Survey of Organizational Functioning and Leadership
SRP: Standard Re-entry Practice
SU: Substance use
SUDs: Substance use disorders
TBRI®: Trust-based Relational Intervention®
TCU: Texas Christian University
TCU DS 5-OS: TCU Drug Screen 5 with Opioid Supplement
TCU THK-NUY: TCU Adolescent Thinking Forms-Negative Urgency Youth Scales
TCU THK- PUY: TCU Adolescent Thinking Forms-Positive Urgency Youth Scales
TLFB: Timeline Followback
